# Medical Organization

**Published:** 1908-06

**Authors:** M. A. Clark

**Affiliations:** Macon, Ga.


### Journal-Record of Medicine

Successor to Atlanta Medical and Surgical Journal, Established 1855, and Southern Medical Record, Established 1870.

**OWNED BY THE ATLANTA MEDICAL JOURNAL CO.**

**Published Monthly.**

*Official Organ Fulton County Medical Society, State Examining Board, Presbyterian Hospital, Etc.*

EDGAR G. BALLENGER, M. D., Editor.
BERNARD WOLFF, M. D., Supervising Editor.
A. W. STIRLING, M. D., C. M., D. P. H., J. S. HURT, B. Ph., M. D., Associate Editors.
E. W. ALLEN, Business Manager.

COLLABORATORS

DR. W. F. WESTMORLAND, General Surgery.
F. W. McRAE, M. D., Abdominal Surgery.
H. F. HARRIS, M. D., Pathology and Bacteriology.
E. B. BLOCK, M. D., Diseases of the Nervous System.
MICHEL HOKE, M. D., Orthopedic Surgery.
CYRUS W. STRICKLER, M. D., Legal Medicine and Medical Legislation.
E. C. DAVIS, A. B., M. D., Obstetrics.
E. G. JONES, A. B., M. D., Gynecology.
R. T. DORSEY, Jr., B. S. M. D., Medicine.
L. M. GAINES, A. B., M. D., Internal Medicine.
J. N. LeCONTE, M. D., Diseases of the Stomach and Intestiness.
L. B. CLARKE, M. D., Pediatrics.
EDGAR PAULIN, M. D., Opsonic Medicine.
R. R. DALY, M. D., Medical Society.
A. W. STIRLING, M. D., Etc., Diseases of the Eye, Ear, Nose and Throat.
BERNARD WOLFF, M. D., Diseases of the Skin.
E. G. BALLENGER, M. D., Diseases of the Genito Urinary Organs.

VOL. X. JUNE, 1908. NO. 3

### MEDICAL ORGANIZATION.*

BY M. A. CLARK, M. D., MACON, GA.

"One of Matthew Arnold's clear thinking Yankees has said with epigrammatic brevity, that whenever three Americans get together, they organize." It is evident that he did not refer to the medical profession.

According to the earliest records, the first association of physicians into a medical society was in Boston in 1735. A report of an operation for stone in the bladder on a boy of six years was made to this society in November, 1741. There is no further record of its existence. The Philadelphia Medical Society was organized in 1765 but enjoyed only a short life.

The oldest medical society existing at the present day was

*Read before the Georgia Medical Association, Fitzgerald, April 15-16-17, 1908.

founded in June 1766 with a membership of sixteen. I refer to the New Jersey Medical Society. Fifteen years later the Massachusetts Medical Society was organized with a membership of thirty.

The first medical organization in the South was in South Carolina, but so far as we can ascertain, it was of short duration. The first permanent society was the Georgia Medical Society in 1804 at Savannah. This society still exists and is one of our best organizations.

Except the Maryland Medical Society, the first state organization in the South was effected in 1846 under the title of the Medical Association of Alabama. This association was reorganized in 1873 so that it should consist of component county societies, but the last county was not organized into a society until 1888. This is the most complete and most powerful medical organization in America, and I do not think that I would exaggerate to say in the world. Fully 92 per cent. of the physicians of the state belong to the society, and through its committees it controls both the Boards of Medical Examiners and of Health, and the county societies are the county boards of health. This is a most striking example of what may be accomplished by the united and persistent efforts of a faithful few.

Packard in his History of Medicine in the United States, says that our own Crawford W. Long presented a full report of his first use of ether as an anesthetic in surgical operations to the Georgia State Medical Society in 1842, but we are unable to find any other reference to such an organization. Our Association was founded in 1849 under the name it now bears, the Medical Association of Georgia, and, as you already know, it was reorganized in 1905.

Under the original plan of organization any reputable physician of the State, when vouched for by two members of the Association and approved by the Board of Censors, became a member of the Association and attended or took part in it as he felt inclined. From year to year, we were able with no little effort to get about enough new members to equal the number of delinquents. Hence, the membership did not increase nor did the interest in the work grow to any extent. At the time of the re-organization in 1905, the total membership was six hundred and twenty-five and there were neither county nor district societies.

The present plan of organization provides that the State Association shall consist of component county societies and the door of entrance must be the county society. In other words the Medical Association of Georgia is an aggregation of county societies and depends wholly upon these societies for its existence. This is a wise provision for there are in every locality matters of interest and importance to the profession of that vicinity which can not be properly dealt with by the State organization meeting annually, but must needs be left to the management of the County society. However, the State society can aid local societies by devising plans and helping them to execute these plans.

The existing plan provides for two bodies, a Legislative and a Scientific. The Legislative body is the business branch of the Association and is composed of delegates from each county society, every society being entitled to at least one delegate, and those having a membership of more than fifty, being allowed a representative for each fifty or fraction thereof. This constitutes the House of Delegates and meets on the afternoon before the Scientific Session sometimes called the General Session. All business and matters pertaining to the good of the profession and the public generally are considered in this body. That we may be democratic in the full sense, the deliberations of this session are submitted to the General Session for its approval. Also, the several delegates on their return to their respective societies should make a complete report of these meetings, that all may feel that they are an integral part of the Association and that it is as much theirs as if they had taken part in the proceedings.

The Scientific or General Session is composed of all members of the county societies in attendance and devotes the three days in session to reading and discussing scientific subjects. Being relieved of the business part of the Association, much more attention is given to the consideration of scientific topics. The short time this plan has been tired fully convinces us of the wisdom of its adoption.

Now, gentlemen, let me remind you, and I beg to make it emphatic, that the county society is the corner stone, the pillars, the whole foundation, yes, the frame work, top, bottom, every part of the Association, and without the county societies there can be no State Association. Whatever affects one affects the other, useless each without the other. I have frequently heard some of the physicians especially of the smaller towns and villages, say that it is well to have a State Society but it is simply impossible to have a county society. We must rid ourselves of this erroneous notion before we can have our organization complete. Just as the brain is composed of multitudinous little cells so arranged that they work in unity, each depending upon and helping the other, and the aggregate of all their work constituting the thought, the will, the mind of the individual; so must the medical men of Georgia make up the working element of our Association, and all dependent upon each other must be so organized that the very best effort of every member is in harmony with that of the other and all together creating the great whole, our beloved Association.

In order that these county societies may be organized and encouraged to live and thrive, it has been deemed wise to have some one whose duty it is to visit them from time to time, at least once yearly, and renew their interest and foster their growth. After mature deliberation, it was thought best to have a representative from each Congressional district to look after the welfare of the counties in his respective district, and the Council was created, consisting of eleven members. They also constitute the Board of Censors and Finance Committee of the Association and are members of the House of Delegates.

The duties of the Councilors are by no means easy. To faithfully fulfill these duties, one must be wise, prudent, tactful, energetic, and ready and willing to make any sacrifice for the good of the profession. It requires a vast deal of thought and study and no little financial loss on account of absence from the duties of home, but the results, the benefits, to the profession and ultimately to humanity, more than repay the true, big-hearted, broad-minded, soul-loving physician. Gentlemen, in choosing your Councilors, you should at all times select the very best men in the Society, those most adapted to the work as well as willing to devote the time and energy to it. Every one, who takes up the work of organizer and does that work well, becomes a better, broader, and more useful man, for he can not organize others without first becoming fully acquainted with and mastering self. No member of this Association should allow himself to seek this high office nor should he ever accept it solely for his own personal aggrandizement.

Fellow members of the Medical Association of Georgia, let me urge you to always select for your Council as well as your other officers the men most adapted to the place and to never allow yourselves to be controlled by personal or selfish motives.

It has been no easy task to organize the county societies we now have, and as has already been said, it is even more arduous to keep them organized and to get them to act. Allow me to recall to you a few of the difficulties as they appeared to me as one of your organizers.

The greatest obstacle to successful organization and the one most difficult to overcome is the petty jealousies among our physicians. How sad but true! Now frequently we find two good men of the profession so jealous of each other that they do not speak to the other in passing but rarely allow an opportunity to pass without saying some little mean thing about the other in the presence of the laity. Some live long and fairly useful lives and die without any fraternal feeling toward their fellow laborers. By thus acting they have made their duties more onerous and have degraded their high calling.

If you please, go to any village or town of the State where there has been no medical society and ask a physician about one of the lawyers or any other layman, and he will gladly recall his virtues and ofttimes magnify them . Now, ask him of his fellow physician and he will either condemn him outright or worse still "damn with faint praise." Is it not strange, passing strange, that such noble, charitable, self-sacrificing men should be so thoughtless of each other and so regardless of the other's welfare? There is not a grander, bigger-hearted, more charitable body of men than the medical men of Georgia, yet how prone at all times to speak slightly of each other and to make insinuating remarks about their brethren, thereby bringing the entire profession into disrepute. We can never command the respect due us by the public so long as we allow this grave fault to be so conspicuous.

Go to the legal fraternity and learn of them, thou medical man! Lawyers often abuse each other in the court room, the one vying with the other in the use of scathing epithets. After the adjournment of court, they go out together laughing and jesting as if nothing unpleasant had occurred. Let a doctor make a slighting remark of another in that august presence and they are henceforth enemies and peradventure may attempt to outdo the notorious representative of pugilistic days. How the laity smile and the tongues of the gossipers wag, and still the doctors go on thus forever.

Now, wherein lies the difference? The lawyer is no better than the doctor. I do not think he is as good. It is because the legal profession are thrown constantly together and are enabled thereby to become well acquainted with their fellows, and there arises a bond of sympathy for each other as a result of this association.

The physician after receiving that priceless piece of sheep skin, alias parchment paper, chooses a location to practice his profession. He is young, hopeful, self-confident, and otherwise proud of self, but distressingly ignorant of the courtesy due from one physician to another, not having been taught on this important subject. Often he has never heard of the Code of Ethics much less learned of its teachings. It is truly the formative period of his professional career, and how he is received by his professional brethren and treated by them, makes or mars a useful life. How often have you heard it said that the old doctor has no sympathy nor respect for the young disciple of Aesculapius; and when we recall some remarks that have emanated from some good old nestors of the profession, we are forced to admit that this must be true. It is no wonder that so many of us draw ourselves up into our little shells and keep so inactive that we are weighted down with the moss of jealousy and hatred and are soured from the lack of communion with our fellows. Too well do I remember the reception accorded me when I first located in a little village of Georgia, and I shudder when I think of the influence it came so near having over me. Fortunately, I began early to attend the Association and was there directed in the proper way and encouraged to cultivate the true professional spirit.

Let me beg you of mature years and of much experience in the vicissitudes of life to extend a helping hand, speak an encouraging word, give a friendly nod in passing, to the young doctor in your midst and above all to refrain from saying anything that will reflect upon him in any way. In helping our younger brethren to get the true conception of the physicians life, we not only help them to success and hapiness but we also benefit our fellows, and incidentally we cause many rays of sunshine to enter into the dark niches of our own lives.

Brethren, when we have freed ourselves from these petty jealousies and despicable bickerings and dwell together in peace and harmony, we will receive the respect and love due our noble calling but so sadly lacking now.

Another drawback to successful organization is procrastination, the sugar coating for laziness. Write to the average doctor to suggest a time to meet, to consider the work of organizing, and where is the answer? The echo answers where but the doctor never. He intends to write but was so busy. If by chance you get his attention long enough to organize, that is the last of it. He really expected to attend the meeting but was busy. We so often delude ourselves with the idea that we are too busy but, if we will only make a beginning, we will be surprised to find that it is so easy to become a constant attendant of these meetings.

These obstacles can be overcome only by having active county societies bringing the members into close and frequent association with each other.

Herbert Spencer has said: "Socially as well an individually, organization is indispensable to growth; beyond a certain point there can not be further growths without further organization." This is equally as applicable to medical men. The county organization means growth of the profession in that county. The convening from time to time and discussing the many phases of our science, the interchange of original ideas, and the relation of personal experience, tend to incite within us new life and greater desires to know more and to do better work. If you know more than the others of your county, come to the society and instruct them; if you know less come and learn of them. You thereby become more learned and correspondingly more skillful. The frequent association in your society the better acquaints you the one with the other. You see the faults of the others and in turn are made conscious of having similar ones; you recognize their virtues, you grow more charitable toward one another; you realize that you are not competitors but are colleagues working together for the good of mankind.

The people notice with wonder this pleasant but unusual relation existing among you, and they appreciate you as never before. You become a potent factor in the affairs of the community in which you live. Your legislators when asked to support certain measures which are to come before that body will not refuse, for they see that you are no longer backbiters and dissenters but are co-workers in one great cause for the good of your fellow man.

It is well to devote occasional meetings to the discussion of the business side of the profession. Having become better physicians, you are entitled to greater compensation. My experience in the work of organization convinces me that it is not best for the profession to have a compulsory free bill, and they are indeed a stumbling block in the way of a complete organization, yet I do think it wise to have a reasonable fee schedule suggestive but not compulsory, and we should discuss the most expedient ways and means of collecting these fees. The majority of the dead beats can be taught to be good pay clients and so much more appreciative, if we will only work together to accomplish that end. Just as long as Dr. A will go to see a patient lest he call Dr. B, and both knowing that the fellow owes Dr. C a large bill of long standing and will not even make an effort to pay it, just that long will our profession continue to be a poorly paid and much imposed upon profession.

The average physician works much more assiduously than he ought to work, and his amiability is spoiled if his life is not shortened by the frequent night calls he is compelled to answer. 99 per cent. of night work is absolutely unnecessary, and if we will only make a united effort, we can soon educate the people that they can wait until a reasonable hour to call a physician. Our evenings are interrupted far more often than necessity requires. We should be permitted to have a little leisure with our families after coming home from a very trying day, too, we need the evenings for study and reflection. The people can be educated to allow this, and we should begin now to do it. Our Sunday work is entirely too great and should be confined to the cases really needing attention on that day. This can be arranged in due time by the proper united effort on our part.

I now wish to call your attention to a few of our needs as regards legislation: We should have a law so defining the practice of medicine as to require the bone rubber, the so-called Christian Scientist, and all such ilk to show equal evidence as we do, of qualification to practice before they are allowed to treat disease.

A law making the communication of the physician and patient privileged, and another defining expert testimony and providing a fee for such testimony, would save us no little embarrasment at times as well as enrich our coffers to some extent. Not a few of us know how delightful it is to have to hang around the court house till the court sees fit to have us on the stand for the lawyer to do his best to either make us appear foolish, or to perjure ourselves, that he may gain his case with the jury.

The State Board of Medical Examiners and the Board of Health should be men selected by us from our Association, and the county boards should be controlled by the county societies.

We are sadly in need of a sanitarium for tuberculosis. Some of us have done excellent work to that end but the legislature apparently ignores us.

Our Boards of Education should have at least one physician on every board, that the proper methods may be adopted for teaching the children how to prevent soil pollution and infection therefrom as well as the dangers of infection from other diseases.

I will not take up more time on this subject though there are other matters of importance that we should consider in due time.

Now, gentlemen, all of these can not be had in a year or even five years, but if we will get our 146 counties so well organized that they will have the desired influence with their legislators, we will accomplish all of this and more.

Just here, let me say that there seems to be a tendency on the part of many to search for new measures to be brought before the legislature, and all of them possess some merit. I earnestly ask that you leave all these alone for awhile and advocate with all your might such laws as are most needed for our welfare and the others will come in due time.

A medium of communication with the members of the Association would facilitate the work of completing our county organizations as well as keep up a renewed interest in the societies. I respectfully recommend that you authorize your Council to enlarge the Bulletin and arrange for the publication from month to month of such matter as will encourage the work of organization. The Bulletin will become a monthly letter from the officers of the Association to the members and will foster the idea that it is the organization of the profession for the benefit of its membership, and each member will feel and show a greater interest in its affairs.

Now, fellow members of the Medical Association of Georgia, while much depends upon our officers as to how successful and useful this organization becomes, yet it can never be complete without the co-operation, support, and sympathy of every individual member. I can not tell you how often your President has yearned for an encouraging word or an expression of sympathy, and I am sure he could have done better work, if you had but thought to offer your sympathy and support. I trust you will go home from this meeting fully resolved to in future give your hand and heart to the perfection of this great work.

Ours is the Empire State of the South, let us make it the banner State of the Union in medical organization.

